# ING3 promotes prostate cancer growth by activating the androgen receptor

**DOI:** 10.1186/s12916-017-0854-0

**Published:** 2017-05-16

**Authors:** Arash Nabbi, Urszula L. McClurg, Subhash Thalappilly, Amal Almami, Mahsa Mobahat, Tarek A. Bismar, Olivier Binda, Karl T. Riabowol

**Affiliations:** 1 Department of Biochemistry & Molecular Biology, Arnie Charbonneau Cancer Institute, Cumming School of Medicine, University of Calgary, Calgary, AB Canada; 2 Department of Oncology, Arnie Charbonneau Cancer Institute, Cumming School of Medicine, University of Calgary, Calgary, AB Canada; 3 Department of Pathology & Laboratory Medicine, Arnie Charbonneau Cancer Institute, Cumming School of Medicine, University of Calgary, Calgary, AB Canada; 4 Solid Tumour Target Discovery Laboratory, Newcastle Cancer Centre, Northern Institute for Cancer Research, Medical School, Newcastle University, Newcastle upon Tyne, UK; 5 Newcastle Cancer Centre at the Northern Institute for Cancer Research, Newcastle University, Paul O’Gorman Building, Medical School, Framlington Place, Newcastle upon Tyne, England NE2 4HH UK; 6 #311 HMRB, 3330 Hospital Dr. NW, Calgary, Alberta T2N 4N1 Canada

**Keywords:** INhibitor of growth 3, ING3, Prostate cancer, Androgen receptor, Prognostic biomarker, Oncogene

## Abstract

**Background:**

The androgen receptor (AR) is a major driver of prostate cancer, and increased AR levels and co-activators of the receptor promote the development of prostate cancer. INhibitor of Growth (ING) proteins target lysine acetyltransferase or lysine deacetylase complexes to the histone H3K4Me3 mark of active transcription, to affect chromatin structure and gene expression. ING3 is a stoichiometric member of the TIP60 lysine acetyltransferase complex implicated in prostate cancer development.

**Methods:**

Biopsies of 265 patients with prostate cancer were stained for ING3, pan-cytokeratin, and DNA. LNCaP and C4-2 androgen-responsive cells were used for in vitro assays including immunoprecipitation, western blotting, Luciferase reporter assay and quantitative polymerase chain reaction. Cell viability and migration assays were performed in prostate cancer cell lines using scrambled siRNA or siRNA targeting ING3.

**Results:**

We find that ING3 levels and AR activity positively correlate in prostate cancer. ING3 potentiates androgen effects, increasing expression of androgen-regulated genes and androgen response element-driven reporters to promote growth and anchorage-independent growth. Conversely, ING3 knockdown inhibits prostate cancer cell growth and invasion. ING3 activates the AR by serving as a scaffold to increase interaction between TIP60 and the AR in the cytoplasm, enhancing receptor acetylation and translocation to the nucleus. Activation is independent of ING3's ability to target the TIP60 complex to H3K4Me3, identifying a previously unknown chromatin-independent cytoplasmic activity for ING3. In agreement with in vitro observations, analysis of The Cancer Genome Atlas (TCGA) data (*n* = 498) and a prostate cancer tissue microarray (*n* = 256) show that ING3 levels are higher in aggressive prostate cancers, with high levels of ING3 predicting shorter patient survival in a low AR subgroup. Including ING3 levels with currently used indicators such as the Gleason score provides more accurate prognosis in primary prostate cancer.

**Conclusions:**

In contrast to the majority of previous reports suggesting tumor suppressive functions in other cancers, our observations identify a clear oncogenic role for ING3, which acts as a co-activator of AR in prostate cancer. Data from TCGA and our previous and current tissue microarrays suggest that ING3 levels correlate with AR levels and that in patients with low levels of the receptor, ING3 level could serve as a useful prognostic biomarker.

**Electronic supplementary material:**

The online version of this article (doi:10.1186/s12916-017-0854-0) contains supplementary material, which is available to authorized users.

## Background

Prostate cancer (PC) is the most frequently occurring male malignancy worldwide. In 2015, more than 220,000 new cases and 27,000 PC-related deaths were reported in the USA [1]. Treatment options include surgery, radiation therapy, and androgen deprivation therapy (ADT). Although most patients initially respond to ADT, they frequently develop recurrent castrate-resistant PC (CRPC) [2] for which management options are limited to aggressive chemotherapy and palliative care.

The androgen receptor (AR) is the central transcription factor in PC biology and pathogenesis. After binding to androgens, the AR translocates to the nucleus, forms homodimers, and binds androgen response elements (AREs) in the promoters of target genes, altering their expression. In primary PC, the inhibition of the AR pathway by anti-androgens leads to dramatic tumor regression. In CRPC, however, tumors acquire resistance to ADT but remain dependent on AR through molecular alterations including AR amplification, mutations, splice variants, as well as overexpression of AR co-activators [3–6]. Co-activators include chaperone proteins, members of the p160 family, DNA repair proteins, ubiquitin ligases, histone demethylases, and acetyltransferases, *inter alia* [7]. Lysine (K) acetyltransferases (KATs) such as p300 and TIP60 have been reported to acetylate and activate the AR in metastatic PC [8–12].

We identified the first member of the INhibitor of Growth (ING) family of epigenetic regulators using PCR-mediated subtractive hybridization between normal and cancerous breast epithelial cells [13], and ING2–5 were subsequently identified by sequence homology [14–16]. ING proteins are stoichiometric members of histone/lysine acetyltransferase (KAT; ING3–5) or histone/lysine deacetylase (KDAC; ING1, ING2) complexes [17]. They specifically recognize H3K4Me3 and recruit KAT or KDAC complexes to alter the chromatin structure [18]. We noted that Yng2, the budding yeast homolog of ING3, is a member of the NuA4 KAT complex, and that deletion of Yng2 caused severe cell cycle and growth defects [19]. Affinity purification followed by mass spectrometry showed that human ING3 is an essential and stoichiometric member of the TIP60 KAT complex that is analogous to NuA4, and its role in this complex is conserved from yeast to mammals [20].

Early studies reported that, similar to ING1, ING3 functions as a type II tumor suppressor to regulate apoptosis and is downregulated in cancers such as melanoma and head and neck carcinoma [15, 21, 22]. However, more recent examination of ING3 function in regulating cardiac hypertrophy indicated a positive growth effect through mTOR [23], and using new, more reliable immunological reagents than were previously available, we found that ING3 is highly expressed in proliferating human tissues such as skin, small intestine, and bone marrow [24].

In a recent screening study, it was noted that high ING3 levels correlate with worse prognosis in patients with erythroblast transformation-specific-related gene (ERG) negative PC, and that a ten-gene signature that correlates with patient survival in these cancers included ING3 [25, 26]. However, the mechanism by which ING3 contributes to ERG-negative PC and its role in PC biology in general were not characterized. Here, we show that ING3 is an AR co-activator, which promotes TIP60-mediated AR acetylation and nuclear translocation, leading to PC cell proliferation and migration. In addition, we provide evidence that ING3 levels correlate with AR levels in PC patient samples, and show that higher ING3 levels serve as a biomarker predicting poorer prognosis in patients with low AR expression.

## Methods

### Cell culture, plasmids, and transfection

LNCaP, VCaP, PC3, DU145, and HEK293T cell lines were purchased from American Type Culture Collection (ATCC), and C4-2 cells were a gift from Dr. Martin Gleave. All lines were periodically checked for mycoplasma by PCR. LNCaP, C4-2, and PC3 were grown in Roswell Park Memorial Institute (RPMI) medium supplemented with 10% fetal bovine serum (FBS). DU145, VCaP, and HEK293T cells were grown in Dulbecco’s modified Eagle’s medium (DMEM) supplemented with 10% FBS. For androgen deprivation, cells were incubated with media supplemented with 5% charcoal stripped FBS (CSS) (Invitrogen) for 48 h. Mibolerone (MB) (Toronto Research Chemicals) was used as an androgen analog at concentrations of 1–10 nM. The pCMV-3myc-AR plasmid was a gift from Dr. Marja Nevalainen. The pCIN4-FLAG-HA-TIP60 was a gift from Dr. Wei Gu. HEK293T cells were transfected using TransIT 293 reagent (Mirus), and PC cells were transfected using Lipofectamine LTX (Invitrogen). Knockdown of ING3 by small interfering ING3 (siING3) was done as previously described [24].

### Lentiviral short hairpin RNA (shRNA) generation and infection

Three shRNA sequences against ING3 (shING3) derived from the RNA interference (RNAi) codex and a scrambled shRNA (shCtrl) with similar GC content were cloned in pINDUCER10, a doxycycline (Dox)-inducible lentiviral vector [27]. C4-2 cells were infected with concentrated inducible lentivirus encoding either shCtrl or shING3. After the addition of Dox, cells were sorted keying on red fluorescent protein (RFP) expression (Additional file 1: Figure S1).

### Immunoprecipitation (IP)

For IP, 1 × 10^7^ cells were lysed at 4 °C using lysis buffer (50 mM Tris-HCL, 150 mM NaCl, 1 mM ethylenediaminetetraacetic acid (EDTA), 1% Triton X-100) supplemented with protease inhibitors (cOmplete, Roche). Antibodies including anti-HA (Roche), anti-Myc (Sigma), anti-acetyl lysine (Santa Cruz Biotechnology), anti-ING3 (Kerafast [24]), anti-AR (N-20, Santa Cruz Biotechnology), or anti-TIP60 (C-7, Santa Cruz Biotechnology) were crosslinked to beads (GE Healthcare) and used for IP.

### In vitro acetylation assays

HEK293T cells were transfected with green fluorescent protein (GFP), ING3-HA, or AR-Myc and were lysed 24 h post-transfection. GFP- or ING3-transfected cell lysates were incubated with anti-HA, and AR-transfected cell lysates were incubated with either anti-Myc or normal rabbit IgG (Santa Cruz). For acetylation assays, IP samples were washed once in histone acetyltransferase (HAT) buffer (50 mM Tris-Cl pH 8.0, 10% glycerol, 0.1 mM EDTA, 10 mM butyric acid, 2 μM TSA). HA-IP samples were mixed with protein A beads or control rabbit IgG IP. The AR-IP sample was divided equally into two tubes and mixed with HA-IP samples from either GFP- or ING3-transfected cell lysates. Following addition of 1 mM acetyl coenzyme A (Lithium salt, Sigma), all tubes were incubated at 30 °C for 1 h with occasional shaking.

### Luciferase reporter assays

We plated 5 × 10^4^ HEK293T cells in 24-well plates and transfected with the indicated plasmids, together with AR3-tkk-LUC (a gift from Dr. Paul Rennie), pCMV-3myc-AR, and a cytomegalovirus (CMV)-beta galactosidase (PBL3-beta-gal) construct as a transfection control (a gift from Dr. Shirin Bonni). Luciferase assays were performed as described. Briefly, one day after transfection, cells were washed with phosphate-buffered saline (PBS) and lysed using reporter lysis buffer (Promega). 30 μl of each lysate was transferred into 96-well plates, and luminescence was detected using a Berthold luminometer. Beta-gal staining was used as an internal control.

### Chromatin immunoprecipitation (ChIP) and quantitative PCR (qPCR)

The effects of ING3 knockdown on AR recruitment to the FKBP5 ARE were determined by ChIP using 3 × 10^7^ C4-2 cells transfected with siCtrl or siING3 for 48 h in media supplemented with 5% CSS, +/– 10 nM MB. Cells were crosslinked using 1% formaldehyde. Cells were lysed in 1 ml ChIP lysis buffer and sonicated for 8 × 12 s. After centrifugation, the supernatants were immunoprecipitated with rabbit anti-AR (N-20, Santa Cruz) or rabbit control IgG overnight at 4 °C and incubated with Protein A Beads (GE Healthcare) for 2 h at 4 °C. Immunoprecipitates were washed with ChIP lysis buffer, with Tris-EDTA (TE) buffer, and then eluted. The IP and input samples were reverse crosslinked using NaCl at 65 °C overnight, and the DNA was isolated. Binding of AR to the Androgen Response Element (ARE) was tested using qPCR. The primer sequences used were FKBP5 ARE6/7: Fwd 5'-CCCCCCTATTTTAATCGGAGTAC-3' and Rev 5'-TTTTGAAGAGCACAGAACACCCT-3', Non-specific Fwd 5'-GGTCAGGTTTTGGTTGAGGA-3' and Rev 5'-CAAGCACAGTGAGGGAGACA-3'. TRIzol and an Omniscript Reverse Transcription kit (Qiagen) were used for isolating total RNA and generating complementary DNA (cDNA). Real-time PCR was performed using Maxima SYBR Green Mastermix (Fermentas) with an Applied Biosystems 7900HT PCR system. The qPCR primer sequences are listed in Additional file 2: Table S1.

### Tissue microarray (TMA) study and quantitative analysis

Sections (4-μm thick) were cut from TMA blocks and deparaffinized in xylene, rinsed in ethanol, and rehydrated. Heat-induced epitope retrieval was at 121 °C, pH 6 in Target Retrieval Solution (Dako) for 3 min in a decloaking chamber (Biocare Medical). Slides were stained using a Dako Autostainer. Endogenous peroxidase activity was quenched with peroxidase block (10 min, Dako) followed by a 15-min protein block (Signal Stain, Cell Signaling, Danvers, MA, USA). Slides were washed with Tris-buffered saline with Tween (TBST) and incubated at room temperature for 60 min with Signal Stain protein block containing a 1:1500 dilution of ING3 mouse mAb [24] and a 1:100 dilution of anti-pan-cytokeratin rabbit polyclonal antibody (Dako). Secondary reagents were incubated at room temperature for 60 min: ready-to-use goat anti-mouse antibody conjugated to a horseradish peroxidase-decorated dextran polymer backbone from the DAKO EnVision + system (Dako) and 1:200 dilution of Alexa-555 conjugated goat anti-rabbit antibody (Invitrogen). Slides were washed with TBST and incubated for 5 min with the Tyramide Signal Amplification (TSA)-Plus Cy5 reagent (Perkin Elmer). After three washes in TBST, slides were mounted with ProLong® Gold anti-fade mounting medium containing DAPI and stored at 4 °C overnight before scanning. For automated image acquisition we used an Aperio Scanscope FL 8/10-bit monochrome TDI line-image capture camera with filters specific for DAPI, Cy3 (Alexa-555) to define the tumor cytosolic compartment based on cytokeratin, and Cy5 for ING3. Images were analyzed using AQUAnalysis® version 2.3.4.1. Scores were based on total percent area positive for ING3.

### Immunofluorescence

Cells were grown on coverslips and fixed with 4% paraformaldehyde in PBS and permeabilized using 0.1% Triton X-100 (Millipore) in PBS. Fixed cells were blocked using 5% BSA for 1 h. Ki67 antibody (Dako) or AR antibody (N-20, Santa Cruz) were used at 1:200 in PBS for 2 h, washed with PBS, and incubated with Alexa-488 goat anti-mouse secondary antibody (1:1000 in PBS/5% bovine serum albumin (BSA)) for 1 h. Cells were then mounted on slides and analyzed using an Axiovert 200 microscope.

### Cell survival and proliferation assay

LNCaP, PC3, and DU145 cells were transfected with siCtrl or siING3 and 1 × 10^4^ cells were seeded in 24-well plates. The cells were washed twice and stained with 0.1% crystal violet for 15 min at room temperature and washed. Alamar Blue assays were performed to estimate cell proliferation according to the manufacturer's protocol. Cell proliferation was also monitored by seeding cells in 96-well plates in parallel and counting cells at the indicated times using a Celigo Cell Cytometer (Cyntellect).

### Anchorage-independent soft agar assay

Agar (0.5%) was prepared in RPMI containing 10% FBS, and 1 ml was poured into each well of 24-well plates to form a bottom layer. 1 × 10^4^ cells were then mixed with RPMI-20% FBS containing 0.3% agarose and poured on top of the bottom layer. Colonies were analyzed using an inverted microscope 10 days after seeding. Colony diameters were measured using ImageJ software, and the colony volumes were calculated (4/3πr^3^).

### Transwell migration assay

Transwell inserts (Corning) were placed in 24-well plates, and 500 μl of RPMI-20% FBS was added to the plate bottoms. We seeded 5 × 10^4^ LNCaP cells on top of the transwell inserts and supplemented with 250 μl of RPMI + CSS or RPMI + 1 nM MB. The inserts were fixed at the indicated time points with 4% paraformaldehyde and methanol and stained with crystal violet. For quantification, six random fields were chosen, and the cells were counted in a single-blinded fashion.

### Wound healing assay

C4-2 cells stably infected with shING3 or shCtrl were plated at 80% confluence in 6-well plates. Doxycycline was added to induce the expression of shRNA, and the cells were grown in RPMI supplemented with 10 nM MB for the duration of the experiment. A 200-μl sterile tip was used to wound the monolayers. At the indicated time points, images were taken from the same fields across the course of the experiment. The percentage of healed wound was then calculated using the following formula: 1-(surface area in one field)/(surface area of the same field at day 0).

### Statistics

All experiments were done in triplicate. Each patient's tissue samples were punched in duplicate on TMA slides. Graphpad Prism was used for graphs and statistical analyses such as standard error calculations, confidence intervals, Student's *t* tests and analysis of variance (ANOVA) statistics. SPSS statistics software was used for analyzing the TMA results including Kaplan-Meier and Cox proportional hazard analyses.

## Results

### ING3 interacts with the AR

**Fig. 1:**
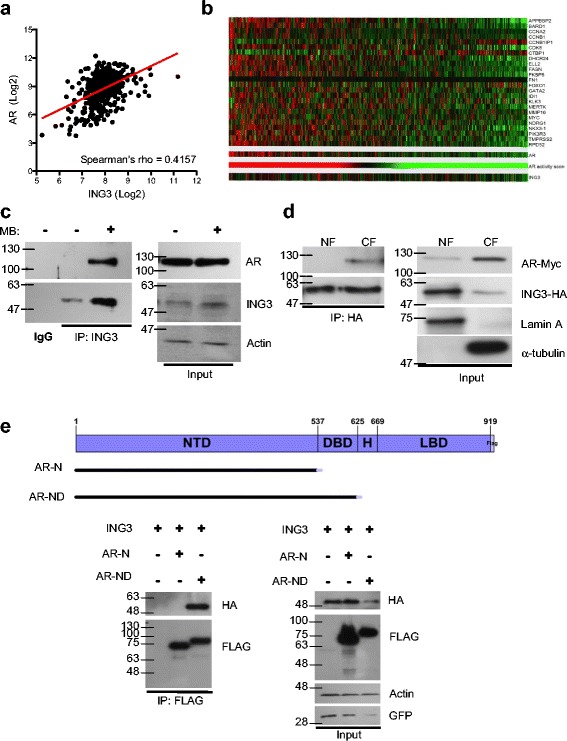
ING3 interacts with the AR. **a** The RNAseq data from TCGA prostate adenocarcinoma cohort (*n* = 498 cancer cases) was retrieved, levels of ING3 and AR mRNA in tumor samples were plotted in log2 scale, and linear regression was graphed. **b** Levels of ING3 and 25 androgen-regulated genes from TCGA RNAseq data were analyzed. AR activity score was generated by summation of z-scores, and patients were organized based on AR activity. **c** Endogenous AR-ING3 interactions were assessed by co-precipitation in LNCaP cells +/– 10 nM MB. **d** HEK293T cells were transfected with ING3-HA and AR plasmids in the absence of MB. Nuclear and cytosolic fractions were isolated according to our Rapid, Efficient, and Practical (REAP) protocol [34] and IPs were performed using HA-bound beads to pull down ING3 in each fraction. The IP samples were then subjected to western blotting by standard methods to detect co-precipitated proteins. Lamin A and α-tubulin were used as nuclear and cytosolic markers, respectively. **e** AR deletion constructs used to map domains required for interaction are shown. Co-precipitation study using HEK293T cells co-transfected with AR deletion mutants and ING3-HA. FLAG beads were used to precipitate AR constructs, and ING3 was detected in IP samples by western blotting

Since ING3 levels regulate PC cell proliferation and correlate with prognosis in patients with ERG-negative PC, we asked whether ING3 had any function in PC cells and whether it interacts with the AR [25, 26, 28]. Correlation analysis using The Cancer Genome Atlas (TCGA) prostate adenocarcinoma cohort data (*n* = 498) indicated that ING3 and AR mRNA levels were positively correlated (Fig. 1a). To test whether this correlation extended to AR function, we analyzed levels of ING3 and 25 common androgen-regulated genes [29, 30]. Heatmaps of patient samples (Fig. 1b) showed that ING3 correlates with overall AR activity score as determined by the 25-gene signature (Spearman rho = 0.21).

VCaP (AR-positive) metastatic PC cells also express higher levels of ING3 compared to LNCaP and C4-2 cells (Additional file 1: Figure S2A, B). To test if ING3 levels correlated with AR activity, these cells were grown in media supplemented with charcoal stripped serum (CSS) for 48 h and treated with the androgen analog mibolerone (MB), the anti-androgen bicalutamide (Bic), or ethanol as a vehicle control. Both AR and ING3 levels increased in response to MB and decreased in response to anti-androgen in VCaP and LNCaP lines. In the C4-2 line, an LNCaP subline of advanced PC with inducible levels of AR and known resistance to anti-androgens, levels of ING3 did not decrease in response to Bic [31, 32] (Additional file 1: Figure S2C). The increase in ING3 protein levels was not a consequence of transcriptional induction (Additional file 1: Figure S2D), but rather ING3 protein was stabilized in LNCaP cells treated with MB where its estimated half-life increased from 1 h to 4 h (Additional file 1: Figure S2E).

A number of co-activators, which interact with and regulate the AR, have been described. To test if ING3 physically interacted with the AR, we immunoprecipitated endogenous ING3 under non-denaturing conditions in LNCaP cells. Probing of blots showed that the AR co-precipitated ING3 in MB-stimulated, but not in unstimulated cells (Fig. 1c). As shown in Additional file 1: Figure S3A, HA-tagged ING3 could co-precipitate Myc-tagged AR in the absence of MB stimulation when overexpressed, but addition of MB increased ING3 levels and co-precipitated more AR. Addition of ethidium bromide (EtBr) to the IP buffer did not alter this interaction, suggesting that the interaction was not dependent on DNA (Additional file 1: Figure S3B) [33].

Nuclear translocation of the AR is a critical step in activation, which involves a series of post-translational modifications and protein interactions. We previously found that ING3 localizes in both the nucleus and the cytoplasm of cells in proliferating tissues [24]. To investigate the subcellular localization of ING3 and the AR, nuclear and cytoplasmic fractions of HEK293T cells transfected with plasmids encoding AR and ING3 in the absence of MB were prepared using the REAP fractionation protocol [34]. ING3 co-immunoprecipitated the AR in the cytoplasmic, but not the nuclear fraction, despite the much higher levels of ING3 in the nucleus (Fig. 1d). To examine the region of the AR required for interaction with ING3, FLAG-tagged deletion constructs of AR were co-transfected with ING3-HA into HEK293T cells. As shown in Fig. 1e, a construct containing the DNA-binding domain (DBD), but not one missing the DBD bound ING3, suggesting that the DBD interacts directly with ING3, or alters the structure of the AR to allow binding of ING3 to the AR. The hinge (H) or ligand-binding domains (LBDs) of the AR did not alter this interaction (data not shown).

### ING3 regulates AR activity

**Fig. 2:**
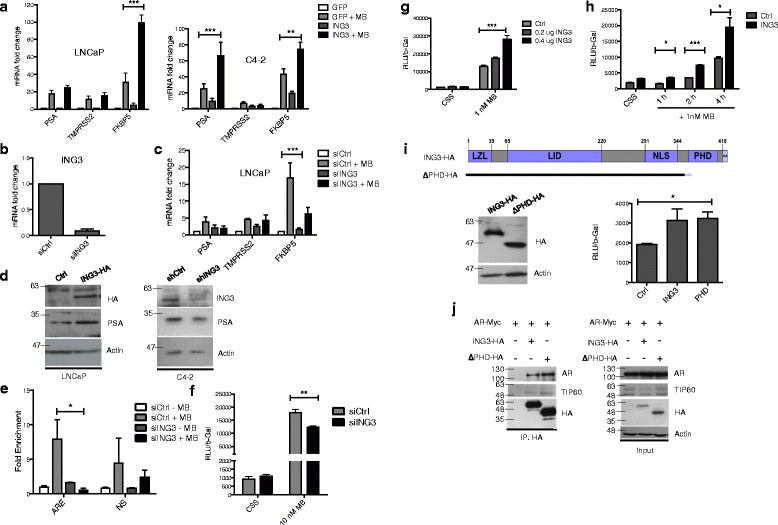
ING3 regulates the AR pathway. **a** LNCaP (*left panel*) or C4-2 (*right panel*) cells transfected with GFP or ING3 constructs were grown for 24 h +/– 10 nM MB and were tested for *PSA*, *TMPRSS2*, and *FKBP5* expression by qRT-PCR (ANOVA **P < 0.01, ***P < 0.001). **b**, **c** LNCaP cells transfected with siCtrl or siING3 for 24 h were treated with 10 nM MB for 24 h. Levels of ING3 (**b**) or androgen-regulated genes (**c**) were assessed by qRT-PCR (ANOVA ***P < 0.001). **d** LNCaP cells transfected with empty vector or ING3-HA were harvested 24 h later, and lysates were blotted with α-HA, α-PSA, or α-actin. Lysates of C4-2 cells transfected with siCtrl or siING3 for 48 h were blotted for the proteins indicated. **e** C4-2 cells transfected with siCtrl or siING3 for 24 h were untreated or treated with 10 nM MB for 24 h. ChIP assays using α-AR antibody and AR-bound DNA used primers specific for an ARE on the *FKBP5* gene (*t* test *P < 0.05). **f** HEK293T cells were co-transfected with 1 μg AR3-tkk-LUC, 0.2 μg β-gal, 0.1 μg GFP, and 20 nM of either siCtrl or siING3 for 48 h +/– MB for the final 24 h. LUC reporter activity was normalized to β-gal (*t* test **P < 0.01). **g** Cells were co-transfected with 1 μg AR3-tkk-LUC, 0.2 μg β-gal, 1 μg AR, and either empty vector or the indicated amounts of ING3 expression plasmid for 48 h +/– MB in the indicated amounts (ANOVA ***P < 0.001). **h** Cells were co-transfected with 1 μg AR3-tkk-LUC, 0.2 μg β-gal, 1 μg AR, and 0.2 μg of either empty vector (Ctrl) or full-length ING3 expression plasmid. 1 nM MB was added for the indicated time points (*t* test *P < 0.05, ***P < 0.001). **i** The map of the plant homeodomain (*PHD*) deleted construct is shown. Cells were co-transfected with 1 μg AR3-tkk-LUC, 0.2 μg β-gal, 1 μg AR, and either empty vector, full-length ING3 expression plasmid, or PHD deletion mutant in CSS medium for 48 h. Levels of ING3-HA expression were verified by western blotting (*left*), and ARE-driven reporter expression is shown in response to full-length and PHD-deleted ING3 (*right*, *t* test *P < 0.05). **j** Co-precipitation using HEK293T cells co-transfected with full-length AR, ING3, and an ING3 deletion mutant. HA beads were used to immunoprecipitate ING3 constructs. The interacting AR and endogenous TIP60 were detected by western blotting with their respective antibodies

To test if ING3 regulated the AR pathway, we overexpressed ING3 in LNCaP and C4-2 cells and analyzed the expression of three androgen-regulated genes, *PSA*, *TMPRSS2*, and *FKBP5*. As seen in Fig. 2a, ING3 potentiated the effects of MB. Conversely, knockdown of ING3 in LNCaP cells (Fig. 2b) decreased responsiveness to MB, affecting *FKBP5* levels most prominently (Fig. 2c). ING3 also increased the levels of *PSA* in LNCaP cells, and ING3 knockdown in C4-2 cells decreased PSA protein levels (Fig. 2d). Since the *FKBP5* gene was regulated most dynamically by ING3 and it functions in AR regulation [35], we tested the effects of ING3 on AR binding to its androgen response element (ARE) in C4-2 cells. MB increased AR binding dramatically, and this increase was blocked by ING3 knockdown (Fig. 2e).

A major function of ING3 is thought to be targeting the TIP60 complex to the H3K4Me3 mark of active chromatin to alter chromatin conformation [17]. We evaluated the effects of ING3 on ARE-driven transcription using a luciferase reporter system [36]. Knockdown of ING3 reduced, but was not able to block the ability of MB to induce luciferase expression driven by ARE (Fig. 2f), while ING3 overexpression enhanced it (Fig. 2g). A time-course experiment with 1 nM MB (Fig. 2h) showed that ING3 affects basal activity of the reporter, and this effect is amplified proportionately over time in the presence of MB.

To further differentiate the chromatin effects of ING3 from its role in the cytoplasm, we performed the luciferase reporter assay using either the wild type or an ING3 PHD deletion mutant. The mutant was capable of stimulating the ARE-driven reporter in the absence of MB to an extent indistinguishable from the wild type (Fig. 2i). This showed that the effects of ING3 on AR transactivation are independent of the ING3 PHD and its chromatin-targeting function. Indeed, the PHD deletion mutant co-precipitated the AR and TIP60 (Fig. 2j), further confirming that the interaction was not dependent on the PHD.

### ING3 promotes activation of the AR by TIP60

**Fig. 3:**
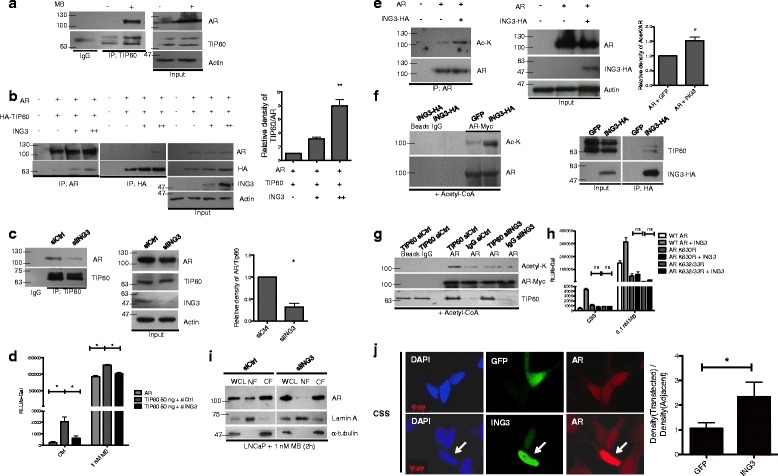
ING3 promotes AR acetylation and regulates its nuclear translocation. **a** TIP60 was immunoprecipitated from C4-2 lysates from cells grown +/– 10 nM MB for 24 h, and lysates were blotted for TIP60 and AR. **b** Cells were co-transfected with AR, TIP60, and increasing amounts of ING3 plasmid + 10 nM MB for 24 h. AR or TIP60 was immunoprecipitated using α-AR or HA-affinity beads, respectively. The graph shows the average ratio of AR:HA-TIP60 in three independent experiments (*t* test **P < 0.01). **c** C4-2 cells transfected with siCtrl or siING3 were treated for 24 h with MB, immunoprecipitated with α-TIP60, and blotted with α-TIP60 or α-AR. The graph shows the average TIP60:AR ratio (*t* test *P < 0.05). **d** ARE reporter activity of cells co-transfected with 1 μg AR3-tkk-LUC, 0.2 μg β-gal, 0.2 μg AR, 50 ng of TIP60, and 20 nM of either siCtrl or siING3 for 48 h +/– 1 nM MB for the final 24 h (ANOVA *P < 0.05). **e** HEK293T cells were co-transfected with 1 μg of vector or AR construct +/– 0.2 μg ING3, and the AR acetylation was determined. The graph shows the average ratio of Ac-AR:total AR (*t* test *P < 0.05). **f**, **g** HEK293T cells were co-transfected with AR-Myc, ING3-HA, or GFP (**f**) or with AR-Myc, siCtrl, or siING3 (**g**). AR was immunoprecipitated with α-myc. ING3 was immunoprecipitated with α-HA in GFP or ING3-transfected cells (**f**). TIP60 was immunoprecipitated in siCtrl- or siING3-transfected cells (**g**). In vitro acetylation assays were performed using 1 mM of acetyl coenzyme A. **h** Cells were co-transfected with 1 μg AR3-tkk-LUC, 0.2 μg β-gal, 0.2 μg of either vector or ING3, and 0.2 μg of either wild-type or mutant AR constructs for 48 h +/– 0.1 nM MB for 24 h. **i** LNCaP cells were transfected with siCtrl or siING3 for 48 h. After 2 h of treatment with MB, nuclear and cytoplasmic fractions were blotted to detect AR. **j** LNCaP cells were transfected with GFP or ING3-HA under androgen deprivation conditions and stained with anti-AR and anti-HA antibodies. The nuclear intensity of AR staining for transfected cells and the adjacent untransfected cells was measured using ImageJ, and the relative ratio was graphed (*t* test *P < 0.05)

Since ING3 is an essential component of the TIP60 complex [20], and TIP60 is a known AR co-activator [10], we asked whether ING3 contributed to TIP60-mediated AR activation. Immunoprecipitates of endogenous TIP60 recovered AR in an MB-sensitive manner as previously reported (Fig. 3a). When cells were co-transfected with increasing amounts of ING3, increasing amounts of AR protein were precipitated with HA-tagged TIP60 and vice versa (Fig. 3b). ING3 increased association between AR and TIP60 by about eightfold (Fig. 3b graph), while siING3 reduced association between AR and TIP60 by about threefold in C4-2 cells (Fig. 3c). An ARE luciferase assay showed that siING3 largely abrogated the effects of increasing TIP60 on AR transactivation in the absence or presence of MB (Fig. 3d), consistent with ING3 facilitating interaction between TIP60 and the AR. These results suggested that ING3 promotes interaction between TIP60 and the AR.

### ING3 promotes AR acetylation and nuclear localization

Since TIP60 acetylates AR and promotes its nuclear translocation [10, 37], we asked whether the ING3 component of the TIP60 complex affects AR acetylation. Overexpression of ING3 increased AR acetylation, as estimated by probing AR immunoprecipitates for acetyl lysine (Fig. 3e). The in vitro acetylation assay shown in Fig. 3f, in which immunoprecipitated ING3-HA was added to AR-IP samples plus acetyl coenzyme A, confirmed that ING3 complexes acetylated the AR. In the complementary experiment shown in Fig. 3g, knockdown of ING3 dramatically decreased the ability of TIP60 to acetylate AR. Transfection with AR acetylation mutants K630R and K632/33R [8] completely abrogated the effect of ING3 compared to transfection with wild-type AR (Fig. 3h).

Knockdown of ING3 also inhibited MB-induced translocation of the AR to the nucleus as seen in the fractionation assay done using the REAP protocol [34] shown in Fig. 3i. Overexpression of ING3 also increased nuclear staining for AR in the absence of MB treatment (Fig. 3j), corroborating previous data indicating a cytoplasmic function for ING3 in promoting TIP60-mediated AR acetylation, leading to its nuclear localization.

### ING3 knockdown reduces PC cell growth

**Fig. 4:**
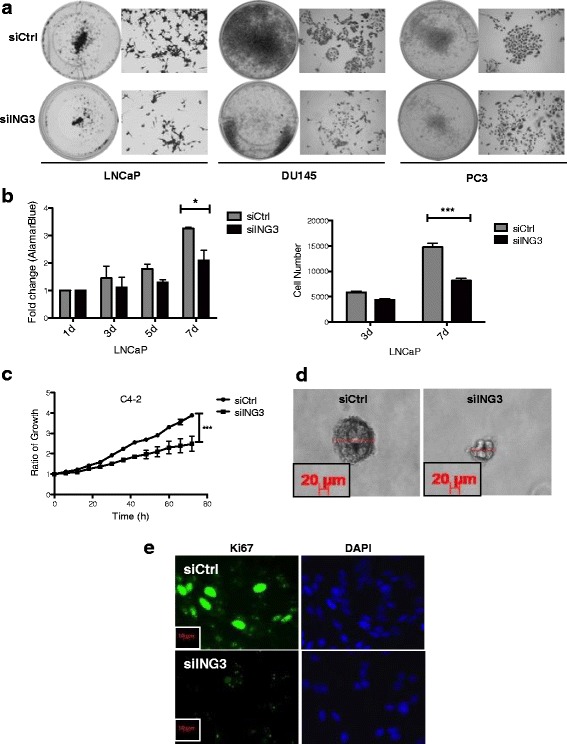
ING3 regulates prostate cancer cell proliferation. **a** LNCaP, DU145, and PC3 were seeded at equal density, transfected with siCtrl or siING3, and 10 days later were fixed and stained with crystal violet. **b** LNCaP cells were grown in media supplemented with 5% CSS for the indicated times, and cellular proliferation was assessed using Alamar Blue (*left*) or counted (*right*) at indicated times (*t* test (*P < 0.05 ***P < 0.001). **c** C4-2 cells were transfected with either siCtrl or siING3 under androgen deprivation conditions and seeded at equal density. Forty-eight h later cell proliferation was analyzed using automated live cell imaging over a 72-h time course (*t* test *P* *** < 0.001). **d** Forty-eight h after transfection with siCtrl or siING3, LNCaP cells were seeded at equal density in 24-well plates in media containing 20% FBS and 0.3% agarose and incubated to allow gels to solidify. Photomicrographs were taken after 10 days, and the diameter of colonies was measured. Representative images of siCtrl and siING3-transfected colonies are shown. **e** Immunofluorescence images of LNCaP cells transfected with either siCtrl or siING3 for 48 h and stained for Ki67 as a proliferation marker

We next investigated the functional consequence of this role of ING3 in PC cells by analyzing the growth of LNCaP, PC3, and DU145 cells after ING3 knockdown. Staining cells 10 days after plating showed that ING3 knockdown decreased proliferation of AR-positive and AR-negative cell lines (Fig. 4a). This observation suggested that ING3 can affect PC cell growth in both AR-dependent and AR-independent manners. ING3 effects on LNCaP (AR-positive) cell growth quantitated using Alamar Blue assays and by counting cells 3 and 7 days post-transfection of siING3 are shown in Fig. 4b, confirming the effects seen in Fig. 4a. Cell growth was also tested using an automated live cell imaging system (IncyCyte Zoom) over a course of 72 h after transfection with siCtrl or siING3. As shown in Fig. 4c, ING3 knockdown also reduced C4-2 cell growth using this independent assay. The colony-forming capability of PC cells in soft agar was also reproducibly reduced upon ING3 knockdown, with average calculated volumes of siING3-transfected colonies being less than half of those for cells transfected with control RNA (313 ± 50 vs. 153 ± 16 mm^3^, Fig. 4d), consistent with cell counting and Alamar Blue assays. LNCaP cells transfected with siING3 also showed significant reduction in Ki67 staining compared to siCtrl, with the sample shown in Fig. 4e further confirming that ING3 knockdown reduced PC cell number by slowing their growth rate. This observation was quantitated using LNCaP cells infected with lentiviral shING3 in which 72% of cells infected with control RFP lentivirus showed Ki67 staining compared to 22% of cells infected with RFP + ING3 lentivirus (Additional file 1: Figure S4).

### ING3 knockdown may reduce migration of PC cells

Transwell migration assays in the absence and presence of MB (Additional file 1: Figure S5A, B) and wound healing assays (Additional file 1: Figure S5C) showed that ING3 knockdown modestly reduced androgen-induced cell migration in LNCaP and C4-2 cells. Consistent with a previous report [25], ING3 also decreased the migration of AR-negative PC3 and DU145 cells (Additional file 1: Figure S5A, B), suggesting that an AR-independent mechanism exists by which ING3 can affect these cells. ING3 knockdown in LNCaP cells also reduced MB-induced filopodia formation as visualized by Alexa 568-conjugated phalloidin staining (Additional file 1: Figure S5D, E), consistent with ING3 having an effect on migration, in addition to effects on cell growth.

### ING3 levels predict survival in patients with PC

**Fig. 5:**
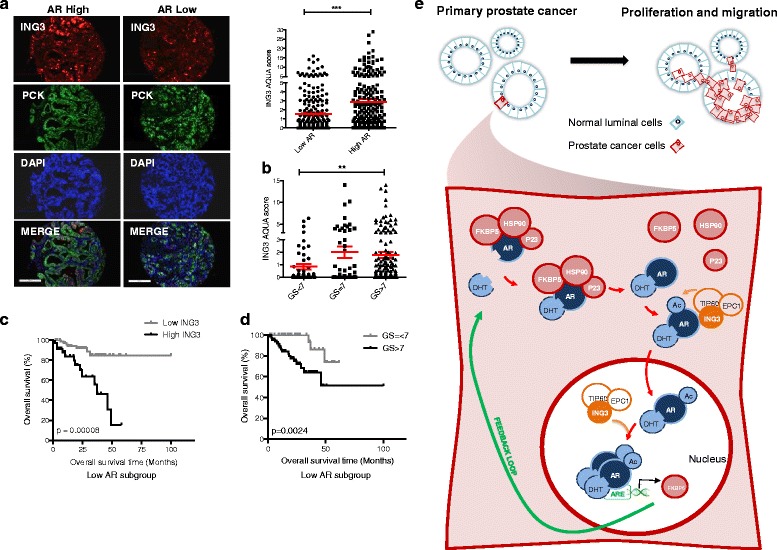
High ING3 levels correlate with poor prognosis in prostate cancer patients. **a** Representative images of patient samples and their ING3 staining (*red*), pan-cytokeratin (*PCK*) (*green*), and DAPI (*blue*) that were classified as having high or low AR levels. The graph shows ING3 AQUA score in the patient subgroups based on their AR expression (Mann-Whitney test ***P < 0.001). **b** ING3 AQUA score in AR-low patient subgroup with various Gleason scores (ANOVA **P < 0.01). **c**, **d** Kaplan-Meier overall survival curves for AR-low subgroup of patients based on ING3 protein levels (**c**) or Gleason score (**d**) (log rank test). **e** Model of ING3 function in the AR pathway. ING3 serves as a scaffolding protein to coordinate interaction between members of TIP60 KAT complex with the AR complex in the cytoplasm. This promotes AR acetylation by TIP60, leading to nuclear translocation. Nuclear AR regulates gene expression promoting prostate cancer survival, proliferation, and migration. ING3 promotes AR recruitment to the FKBP5 ARE (and a subset of other genes containing AREs) and increases FKBP5 mRNA levels. This generates a positive feedback loop, making more FKBP5 available to bind AR, thereby stabilizing the complex, which promotes additional binding to ligand. Activation of this pathway promotes proliferation and migration of AR-responsive prostate cancer cells

Microarray slides containing 265 PC patient tissue samples were stained with a validated ING3 monoclonal antibody [24] and analyzed by automated quantitative immunofluorescence (AQUA) using a blind experimental protocol. Patient characteristics are shown in Additional file 2: Table S2 with representative images of AR staining in Additional file 1: Figure S6. As shown in Fig. 5a, cells with high AR levels stained more intensely for ING3. Examination of our PC cohort indicated a trend of higher ING3 levels in samples with higher (7 or above) Gleason scores compared with samples with Gleason scores below 7 (Fig. 5b), and similar results were seen using TCGA prostate cohort data (not shown). To determine if ING3 had prognostic value in PC, we split the cohort into derivation and validation datasets [38]. Additional file 2: Table S2 shows characteristics of the datasets validated by Kaplan-Meier analysis using the Gleason score as the known predictor (Additional file 1: Figure S7). ING3 AQUA scores were dichotomized using a 1.66 cutoff based on the derivation dataset for testing in the validation dataset. Kaplan-Meier analysis based on AR status showed that higher ING3 levels inversely correlated with overall survival in patients with low AR levels (*P* = 0.00008) (Fig. 5c and Additional file 1: Figure S8). In patients with low AR, ING3 predicted survival better than Gleason score (compare Fig. 5c and d), but Gleason score predicted survival better in patients with high AR levels (data not shown). This suggests that under low AR levels overexpression of ING3 activates the AR pathway, while in the context of higher AR expression, ING3 may not be required for sufficient AR activity to drive cell growth. ING3 levels were also useful in predicting the hazard function using Cox proportional hazard analysis [39]. Factors such as AR levels, age, Gleason score, occurrence of CRPC, and ERG were taken into consideration in the multivariate analyses (Table 1 and Additional file 2: Tables S3 and S4). The contribution of ING3 in predicting hazard function was significant in both tested datasets with hazard ratios of 3.309 and 2.571, respectively. Testing the regression coefficients of Cox models on the two datasets indicated that ING3 coefficients were not significantly different, implying that ING3 contributes similarly to the outcome prediction and was independent of patient dataset.

**Table 1:** Cox proportional hazard model for prostate adenocarcinoma (PCA) cohort

Covariate	Coefficient	SE	*P* value	Hazard ratio	95% CI
ING3	0.887	0.337	0.009	2.429	1.254–4.705
Age	-0.662	0.387	0.725	0.993	0.956–1.032
CRPC	1.385	0.355	0.000096	3.995	1.992–8.012
Gleason score	1.803	0.503	0.0003	6.069	2.264–16.272
AR expression	-0.206	0.334	0.537	0.814	0.423–1.566
ERG expression	-0.007	0.019	0.087	0.516	0.242–1.1

Perhaps more clinically relevant, when Cox regression analyses +/–ING3 were performed and the likelihood ratios (LRs) compared, ING3 significantly improved the Cox model in prediction of the hazard function (ΔLR = 5.075, *P* value = 0.024, ΔLR = 3.941, *P* value = 0.047 for derivation and validation datasets, respectively). This set of results suggests that ING3 could serve as a novel prognostic factor in PC pathophysiology to help predict the aggressiveness of the tumor, which should reduce the rate of overdiagnosis in this patient population.

## Discussion

The androgen receptor (AR) pathway is a major contributor to prostate cancer and, coupled with other oncogenic signaling pathways, plays a key role in the initiation and progression of this disease [40, 41]. In this study, we have identified ING3 as a novel AR co-activator in PC. Altering ING3 levels in PC cells showed that it positively regulates AR, enhancing effects of androgens on the expression of AR-regulated genes and an ARE-driven reporter. ING3 exerts this effect by promoting AR-TIP60 interaction, thereby increasing the acetylation of AR, its translocation to the nucleus, and activation as a transcription factor. This does not require the ING3 PHD region that interacts specifically with the H3K4Me3 [19, 20, 42], identifying a novel chromatin-independent role of ING3. Moreover, knockdown of ING3 inhibits PC cell growth, indicating that ING3 plays an oncogenic role in prostate cancer. In contrast to previous studies in other tumor types where ING3 was reported to function as a tumor suppressor, we find that ING3 levels are higher in aggressive PCs, and that a high level of ING3 is a prognostic factor predicting poorer survival in patients with low AR levels. A model for how our data suggest that ING3 functions to activate the AR signaling pathway in PC biology is shown in Fig. 5e.

A carboxyl-terminal deletion mutant of ING3 lacking the PHD (Fig. 2i) interacted with and efficiently activated the AR. This function is likely distinct from epigenetic properties of ING3 since this form of ING3 is incapable of targeting the TIP60 complex to the H3K4Me3 mark. Similar PHD-independent effects for ING family proteins were seen for ING2 during C2C12 myoblast differentiation [43] and ING4-induced apoptosis in prostate epithelium [44]. These data indicate two distinct functions for ING3, one in coordinating a cytoplasmic complex to enhance AR acetylation efficiency and another to target the TIP60 complex to chromatin via recognition of H3K4Me3. Altered localization of ING1 in brain cancers [45] and its shuttling from the cytoplasm to nucleus by interaction with 14-3-3 proteins [46] are consistent with ING proteins functioning in multiple cell compartments.

Recently, the PHD of ING3 was reported to be essential for DNA damage-induced apoptosis in MCF-7 cells where interaction between the ING3 PHD and the H3K4Me3 mark was reported to be in the submicromolar range [47]. In contrast to this role, we have identified a chromatin-independent function for both full-length ING3 and a shorter isoform lacking the C-terminal domain. These differential functions of ING3 isoforms are not well defined and require further investigation. Consistent with our observation that ING3 interacts with and targets the TIP60 KAT complex to activate the AR by acetylation, a recent study reported that ING1, a stoichiometric member of the Sin3A KDAC complex [17] that directs deacetylation activity, functions as an AR co-repressor [48]. These and other studies, therefore, support the idea that the ING proteins can function to target acetylation and deacetylation activities to the H3K4Me3 mark in chromatin, as well as serve as scaffolding proteins to promote the acetylation or deacetylation of target proteins in the cytoplasm that have major impact on biological processes such as the AR pathway.

AR acetylation is known to be an essential step in AR activation, and increased activity of KATs such as p300 and TIP60 is involved in progression of PC [8–10]. This post-translational modification occurs in the hinge region of AR, leading to nuclear localization signal (NLS) unmasking and nuclear translocation. ING3 promoted TIP60-AR association in the cytoplasm, inducing AR acetylation, nuclear translocation, and activation of target genes, including *FKBP5*, an immunophilin that regulates the AR, NF-kB, and the glucocorticoid receptor [49]. *FKBP5*, induced by AR via several AREs, modulates the AR pathway through forming a positive feedback loop [35, 50, 51] as noted in Fig. 5e. While we observed ING3 effects on AR binding to the *FKBP5*-ARE, the role of other pathways in regulation of this and other AR-sensitive genes cannot be excluded. The differential effects of ING3 on *FKBP5* as well as other selected androgen-regulated genes in this study underline the complex nature of the regulation of these genes. Indeed, according to the ENCODE data portal, there are several other transcription factors that can occupy the promoters of *PSA*, *TMPRSS2*, and *FKBP5*, the list of which interestingly includes (but is not limited to) other members of the nuclear receptor family. It is therefore likely that the overall effect of ING3 on selected genes can be due to its differential regulation of other transcription factors, independent of its AR-activating function. The interaction of ING3 with the DNA-binding domain of the AR, which is the most conserved domain among nuclear receptors, further suggests other functions beyond the AR; these remain unclear at this point and require additional investigation. This interaction can also be of clinical relevance in prostate cancer, as AR splice variants that lack an intact DNA-binding domain are described as one of the mechanisms promoting CRPC [52].

When PC cells were grown in an androgen-depleted medium that mimics clinical androgen deprivation therapy (ADT) conditions, ING3 knockdown significantly reduced cell growth. Consistent with this observation, an independent RNA interference-based screen identified ING3 as a positive regulator of proliferation and survival in androgen-deprived VCaP cells [28]. This is of clinical significance, as resistance to ADT is one of the important challenges in PC management. However, ING3 knockdown also reduced the growth and migration of AR-negative prostate cancer cells, DU145 and PC3, indicating its role in AR-independent pathways. This is likely considering the fact that ING3 and its associated KAT complex are epigenetic regulators with diverse cellular functions [53]. While we report the novel role of ING3 in AR pathway activation, the effects on global gene regulation and chromatin remodeling, as expected from this class of proteins, should not be overlooked. Indeed, our preliminary observations suggest that ING3 can affect cell cycle pathways through its chromatin binding properties.

In contrast to previous studies in melanoma and hepatocellular carcinoma reporting reduced ING3 levels in aggressive cancers [54], our analyses of primary prostate tumors showed that high levels of ING3 predict poorer outcome in patients with low AR levels. A similar trend was observed when analyzing recurrence rate using TCGA data stratified based on AR levels (data not shown). This indicates that higher ING3 levels can compensate for low AR levels by activating AR, promoting PC growth. In the context of AR hyperactivation, ING3 may not be required in the process and may primarily function through gene regulation, for example, by reducing apoptosis upon RSK-mediated suppression [55]. In addition to the effects of androgens and AR antagonists, epithelial-mesenchymal transition (EMT) and invasion of PC cells were recently reported to be dependent on the levels of AR protein, with low levels of AR promoting androgen-induced EMT [56]. This is in line with our findings that ING3 modulated cell migration and that higher ING3 levels correlated with poorer outcome in the subset of patients having tumors with low levels of AR. We recently found that high levels of ING3 also correlate with poorer survival in ERG-negative PC [25]. Although the interplay between ERG and AR remains unclear, several studies have suggested that the ERG fusion protein inhibits AR expression and activity at several loci [57], supporting the idea that ING3 can potentiate the activity of the AR pathway, particularly when AR inhibitory factors such as ERG are absent. Together, these data identify ING3 as a proto-oncogene in PC by regulating the AR pathway through acetylation, and identify it as a novel prognostic biomarker for primary prostate cancer.

## Conclusions

In this study we show that ING3 regulates the AR pathway in prostate cancer by virtue of acting as a scaffolding component of the TIP60 complex, promoting AR acetylation, its nuclear translocation, and the activation of androgen-responsive genes. This study has also validated ING3 as a novel prognostic biomarker that can dramatically improve prediction of overall survival in prostate cancer, particularly in cases with low levels of AR.

## Additional files

Additional file 1: Figure S1. The four panels show representative images of a line of C4-2 cells stably infected with inducible lentiviral shCtrl or shING3, with or without Dox. The western blots show the efficiency of ING3 knockdown in the presence or absence of Dox. **Figure S2**. (A) Lysates from three AR-positive prostate cancer cell lines were subject to western blotting with antibodies against ING3, GAPDH, and actin. (B) mRNA levels of ING3 were normalized to actin in three prostate cancer cell lines. (C) LNCaP, C4-2, and VCaP cells were grown in media with charcoal stripped serum (*CSS*) for 48 h and treated with mibolerone (*MB*) or bicalutamide (*Bic*). Protein levels of ING3 and AR were visualized by western blotting with actin used as a loading control. (D) qRT-PCR study of ING3 in LNCaP cells after treatment with increasing concentrations of MB. The *left graph* shows mRNA levels of ING3 in response to MB. The *right graph* shows mRNA levels of seven androgen-regulated genes in response to MB. (E) A cycloheximide experiment using LNCaP cells grown in the presence or absence of MB to estimate ING3 half-life. **Figure S3.** (A) HEK293T cells were co-transfected with 1 μg of Myc-tagged AR and 1 μg of either empty vector or HA-tagged ING3 +/– 10 nM MB. ING3 was pulled down with HA-affinity beads, and precipitates were blotted with α-AR and α-HA. (B) To determine the effects of DNA on the interaction, co-immunoprecipitations were repeated with addition of ethidium bromide (*EtBr*). ING3 was precipitated using HA-affinity beads. **Figure S4.** LNCaP cells were infected with shCtrl or shING3 lentiviral particles for 72h under androgen deprived conditions and stained with anti-Ki67. Arrows indicate infected (RFP-positive) cells with associated Ki67 staining. RFP-positive and Ki67-positive cells were counted and percentages are shown in the table. **Figure S5.** ING3 affects PC migration. (A) LNCaP, PC3, and DU145 cells were transfected with either siCtrl or siING3 and, in case of LNCaP, treated with 1 nM MB for the times indicated. Transwell migration assays were performed at the indicated time points. Representative images are shown. (B) Images were taken from six random fields for each condition and counted manually on a computer using a blind experimental protocol (*t* test * < 0.05, ** < 0.01). (C) Wounds were made in monolayers of C4-2 cells stably expressing either shCtrl or shING3 in the presence of 10 nM MB and Dox to induce shRNA expression. Wounds were then allowed to heal during a course of 4 days. Images were taken from the same fields for each condition. Percentage of healed wound was then calculated based on pixels observed in each condition. (D) LNCaP cells were transfected with siCtrl or siING3 and treated with 1 nM MB for 72 h, then fixed and stained with Texas Red-conjugated phalloidin. *Arrows* highlight actin projections along cell axes consistent with filopodia formation. (E) The numbers of actin projections per cell were counted in a blind experimental protocol from a total of 50 cells, and the mean number of filopodia/cell was plotted (*t* test *** < 0.001). **Figure S6.** Representative images of prostate cancer samples showing low and high expression of AR as determined by immunohistochemistry. Samples are from the prostate cancer patient cohort used in this study. **Figure S7.** Kaplan-Meier survival curves using Gleason score as a known prognostic marker in the derivation and validation datasets. **Figure S8.** Kaplan-Meier survival curves in our prostate cancer patient cohort with low levels of AR, in the derivation and validation datasets. (PPT 4506 kb)

Additional file 2: **Table S1.** List of primers for qPCR experiments. **Table S2.** Patient characteristics in prostate cancer cohort and the derived datasets. **Table S3.** Cox proportional hazard model for derivation dataset. **Table S4.** Cox proportional hazard model for validation dataset. (DOCX 115 kb)
