# Supplementary material for: ING3 promotes prostate cancer growth by activating the androgen receptor
Source: BMC Med. 2017 May 16;15:103. doi: 10.1186/s12916-017-0854-0 (PMC5434536; doi:10.1186/s12916-017-0854-0)
Supplement: Supplementary file 1 — The four panels show representative images of a line of C4-2 cells stably infected with inducible lentiviral shCtrl or shING3, with or without Dox. The western blots show the efficiency of ING3 knockdown in the presence or absence of Dox. Figure S2. (A) Lysates from three AR-positive prostate cancer cell lines were subject to western blotting with antibodies against ING3, GAPDH, and actin. (B) mRNA levels of ING3 were normalized to actin in three prostate cancer cell lines. (C) LNCaP, C4-2, and VCaP cells were grown in media with charcoal stripped serum (CSS) for 48 h and treated with mibolerone (MB) or bicalutamide (Bic). Protein levels of ING3 and AR were visualized by western blotting with actin used as a loading control. (D) qRT-PCR study of ING3 in LNCaP cells after treatment with increasing concentrations of MB. The left graph shows mRNA levels of ING3 in response to MB. The right graph shows mRNA levels of seven androgen-regulated genes in response to MB. (E) A cycloheximide experiment using LNCaP cells grown in the presence or absence of MB to estimate ING3 half-life. Figure S3. (A) HEK293T cells were co-transfected with 1 μg of Myc-tagged AR and 1 μg of either empty vector or HA-tagged ING3 +/– 10 nM MB. ING3 was pulled down with HA-affinity beads, and precipitates were blotted with α-AR and α-HA. (B) To determine the effects of DNA on the interaction, co-immunoprecipitations were repeated with addition of ethidium bromide (EtBr). ING3 was precipitated using HA-affinity beads. Figure S4. LNCaP cells were infected with shCtrl or shING3 lentiviral particles for 72h under androgen deprived conditions and stained with anti-Ki67. Arrows indicate infected (RFP-positive) cells with associated Ki67 staining. RFP-positive and Ki67-positive cells were counted and percentages are shown in the table. Figure S5. ING3 affects PC migration. (A) LNCaP, PC3, and DU145 cells were transfected with either siCtrl or siING3 and, in case of LNCaP, treated with 1 nM MB for the tim [file 12916_2017_854_MOESM1_ESM.ppt]

## Slide 1
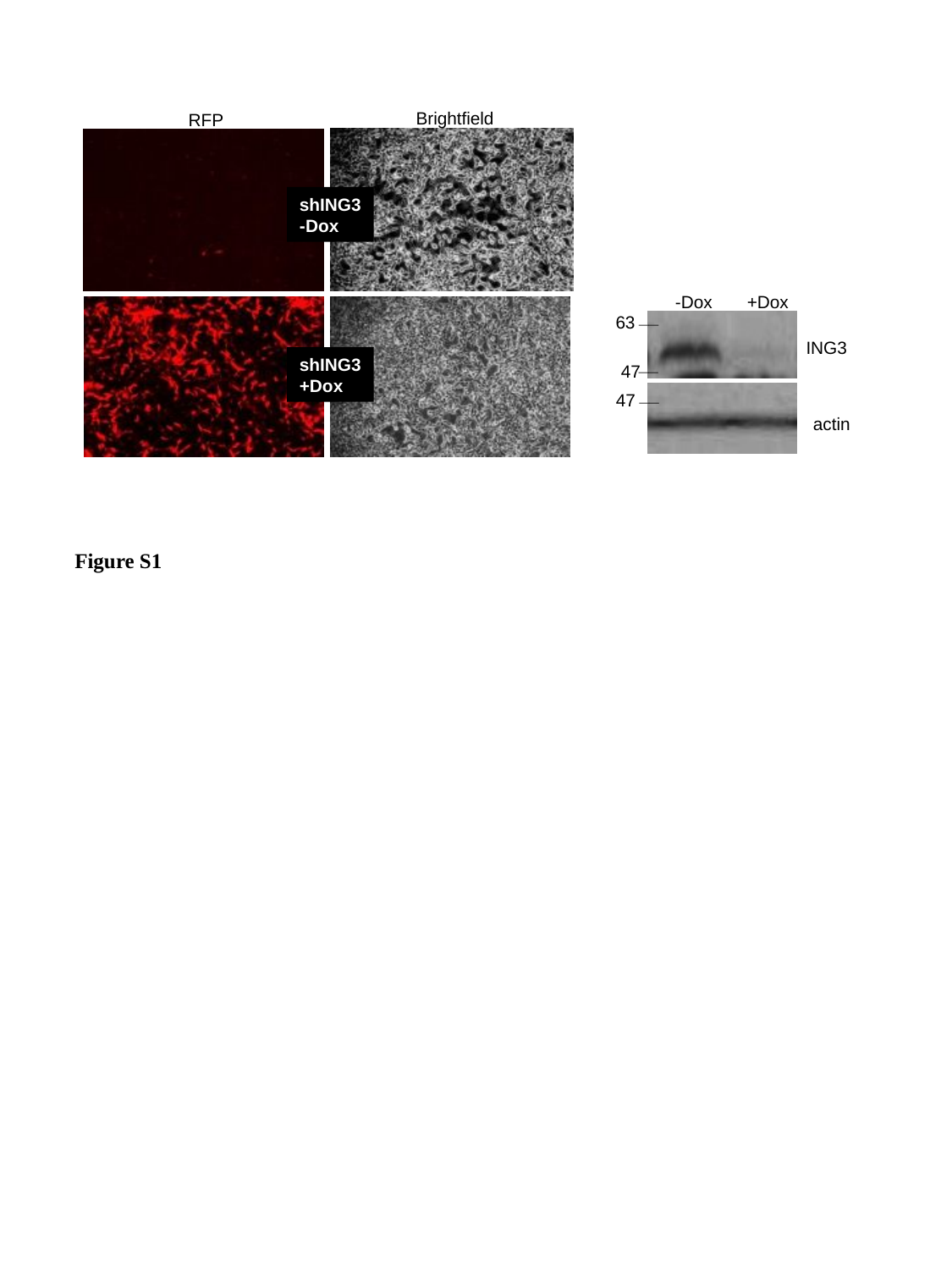

Brightfield
RFP
shING3
-Dox
shING3
+Dox
+Dox
-Dox
__
63
ING3
__
47
__
47
actin
Figure S1

## Slide 2
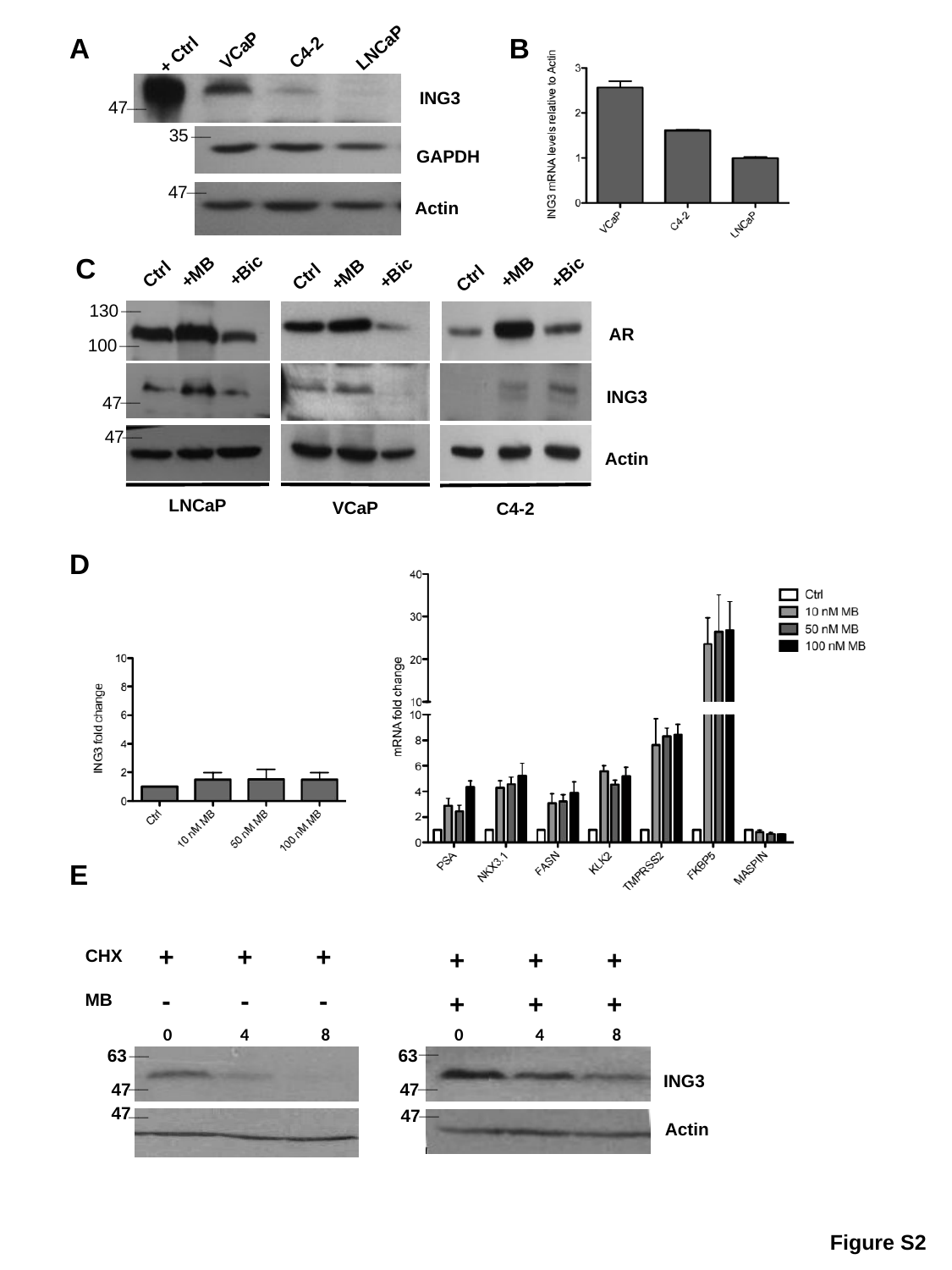

LNCaP
VCaP
C4-2
+ Ctrl
ING3
__
47
__
35
GAPDH
__
47
Actin
A
B
C
+Bic
+MB
+MB
+Bic
+Bic
Ctrl
+MB
Ctrl
Ctrl
__
130
AR
__
100
__
ING3
47
__
47
Actin
LNCaP
VCaP
C4-2
D
E
| + | + | + |
| --- | --- | --- |
| - | - | - |
CHX
| + | + | + |
| --- | --- | --- |
| + | + | + |
MB
0 4 8 0 4 8
__
__
63
63
ING3
__
__
47
47
__
__
47
47
Actin
Figure S2

## Slide 3
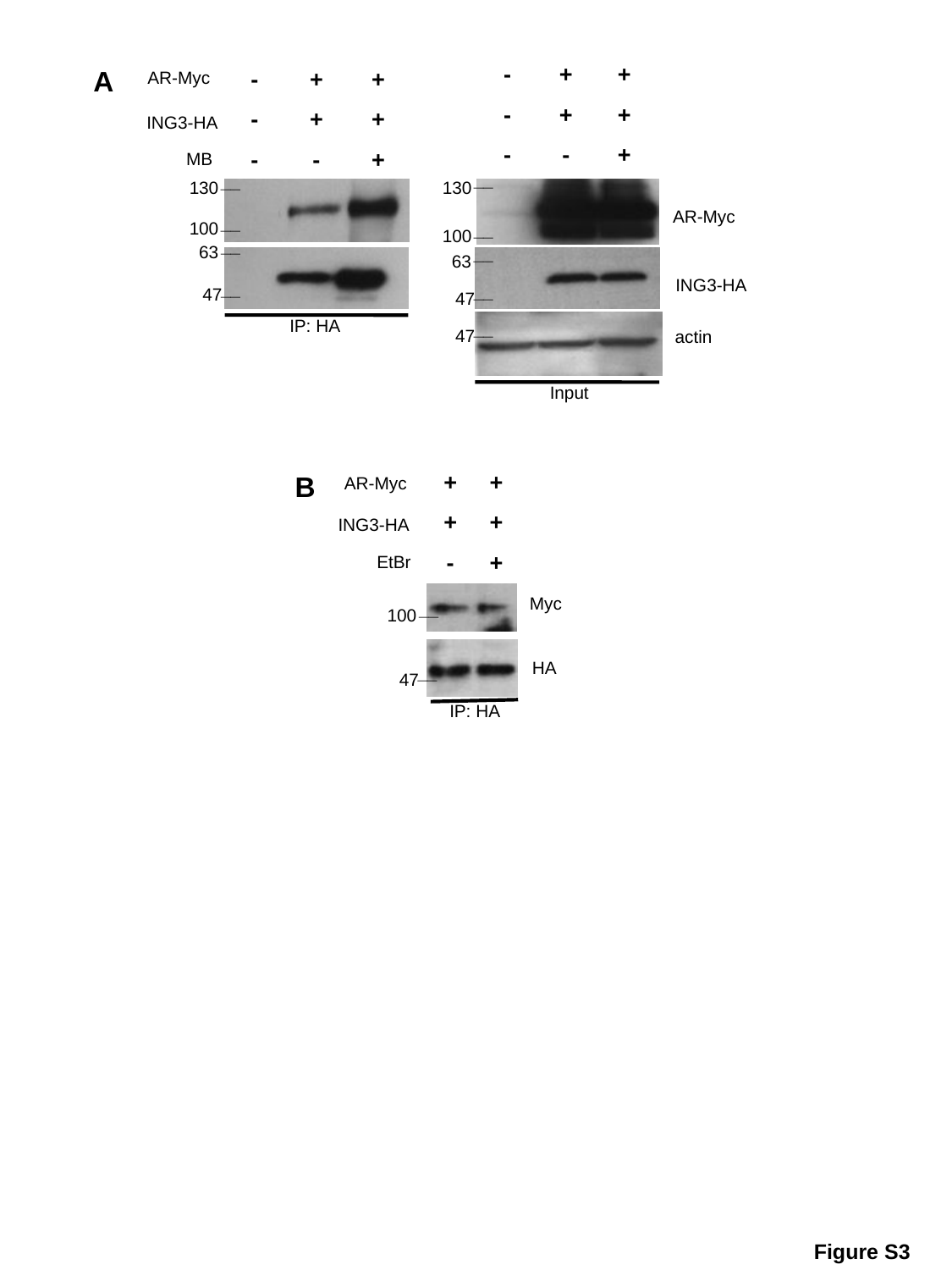

| - | + | + |
| --- | --- | --- |
| - | + | + |
| - | - | + |
A
AR-Myc
| - | + | + |
| --- | --- | --- |
| - | + | + |
| - | - | + |
ING3-HA
MB
__
__
130
130
AR-Myc
__
100
__
100
__
63
__
63
ING3-HA
__
__
47
47
IP: HA
__
47
actin
Input
B
| + | + |
| --- | --- |
| + | + |
| - | + |
AR-Myc
ING3-HA
EtBr
Myc
__
100
HA
__
47
IP: HA
Figure S3

## Slide 4
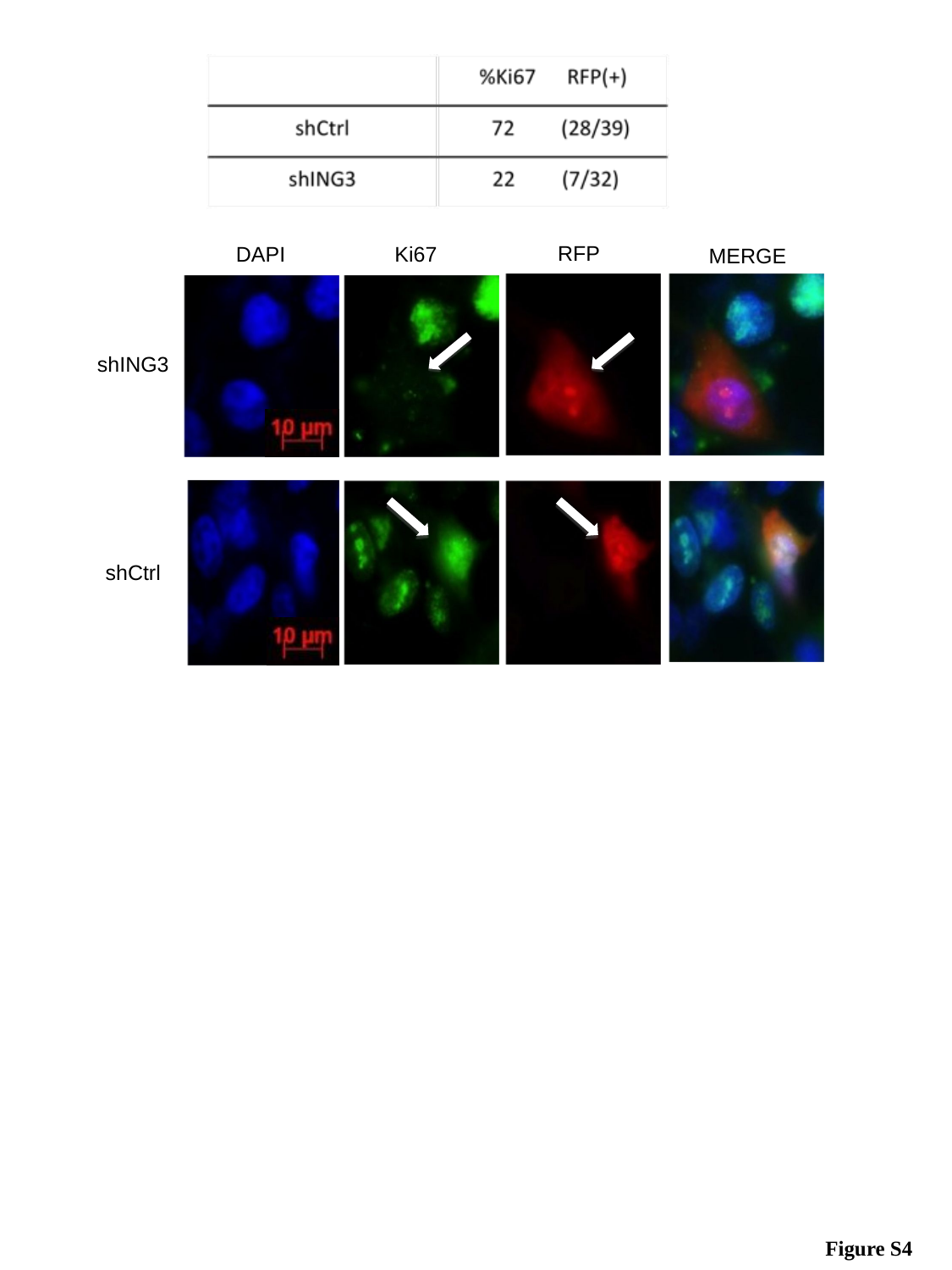

RFP
DAPI
Ki67
MERGE
shING3
shCtrl
Figure S4

## Slide 5
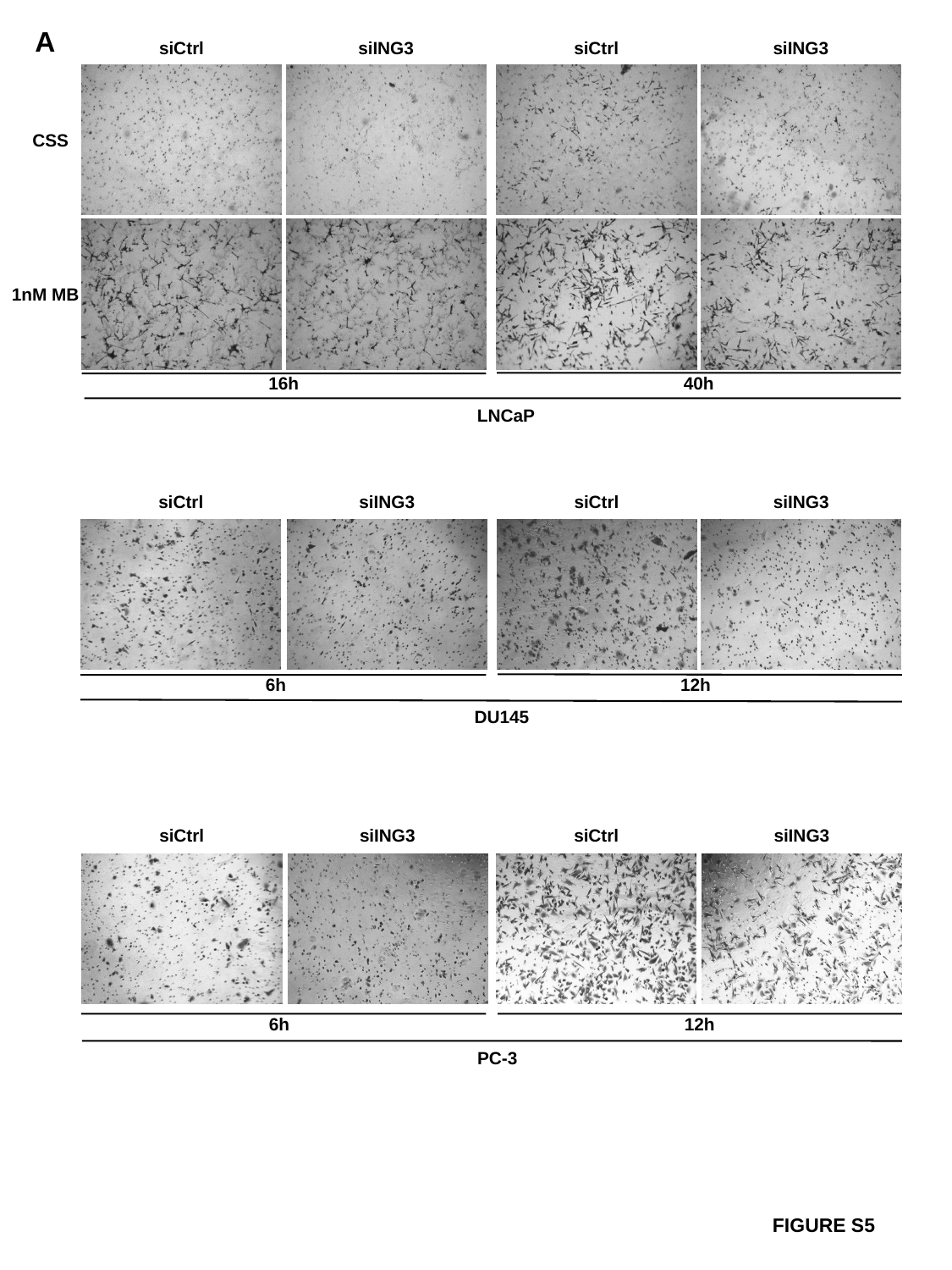

A
siCtrl
siING3
siCtrl
siING3
CSS
1nM MB
16h
40h
LNCaP
siCtrl
siING3
siCtrl
siING3
6h
12h
DU145
siCtrl
siING3
siCtrl
siING3
6h
12h
PC-3
FIGURE S5

## Slide 6
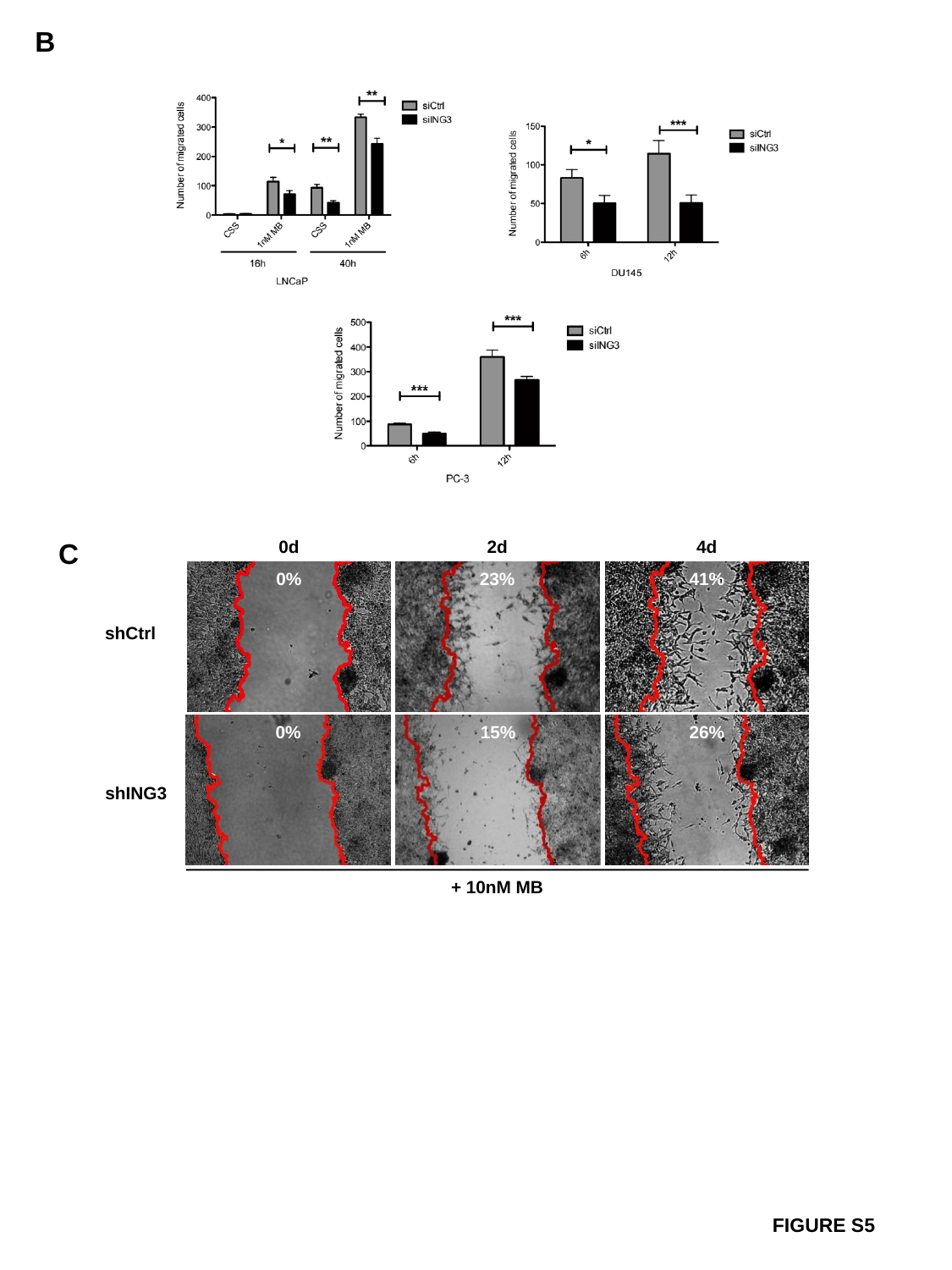

B
C
0d
2d
4d
0%
23%
41%
shCtrl
0%
15%
26%
shING3
+ 10nM MB
FIGURE S5

## Slide 7
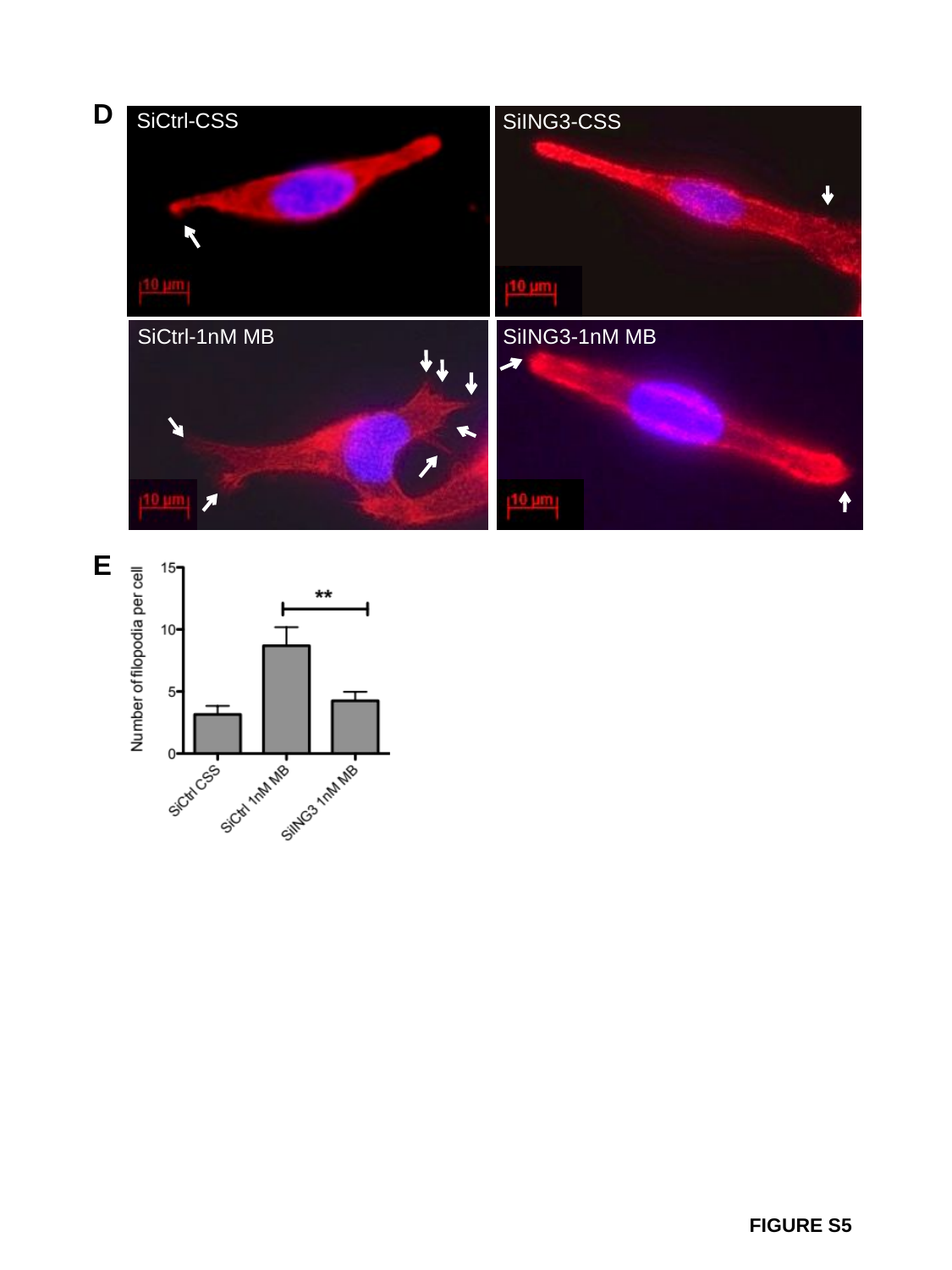

D
SiCtrl-CSS
SiING3-CSS
SiCtrl-1nM MB
SiING3-1nM MB
E
FIGURE S5

## Slide 8
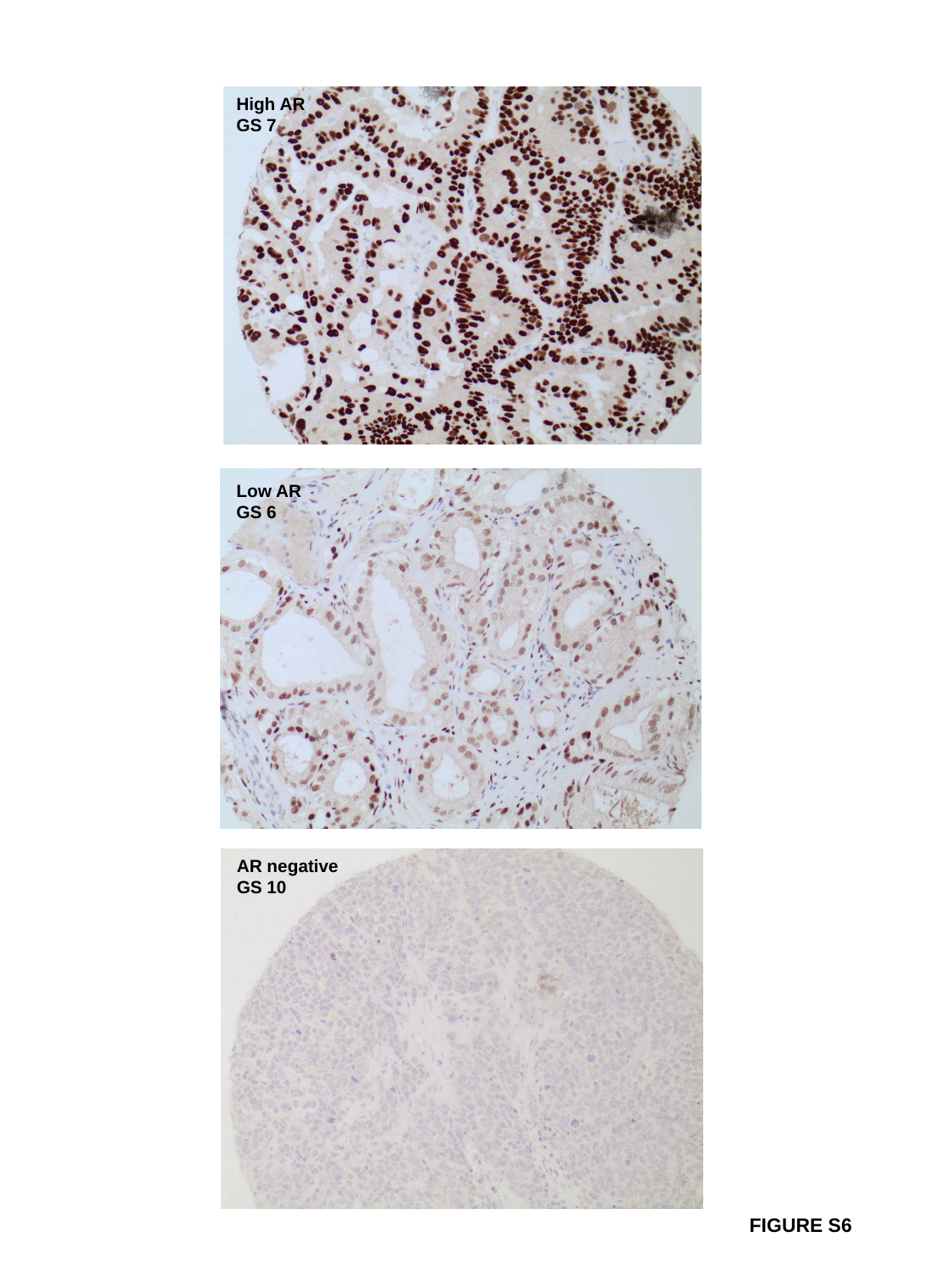

High AR
GS 7
Low AR
GS 6
AR negative
GS 10
FIGURE S6

## Slide 9
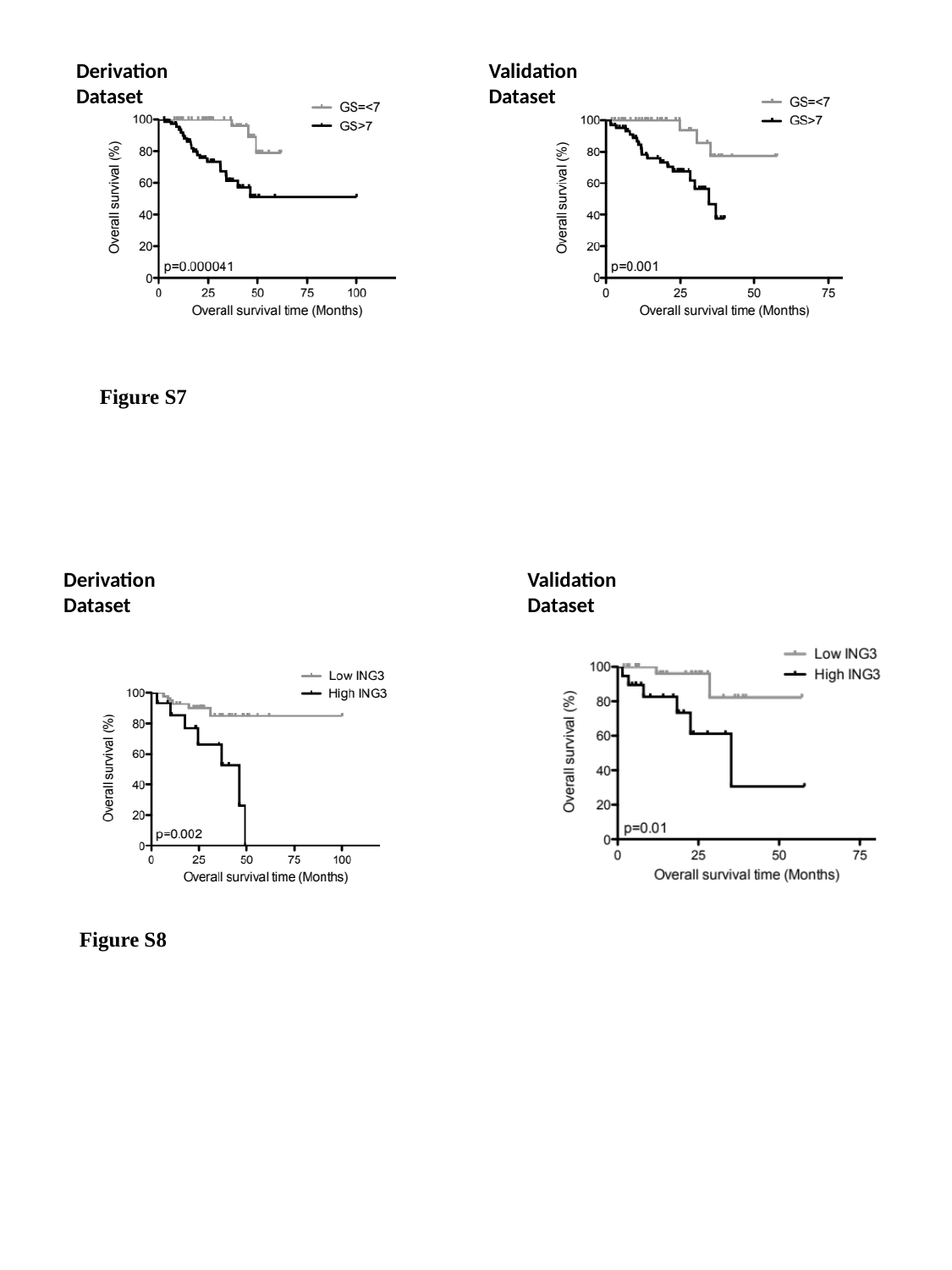

Derivation
Dataset
Validation
Dataset
Figure S7
Derivation
Dataset
Validation
Dataset
Figure S8
