# Supplementary material for: ING3 promotes prostate cancer growth by activating the androgen receptor
Source: BMC Med. 2017 May 16;15:103. doi: 10.1186/s12916-017-0854-0 (PMC5434536; doi:10.1186/s12916-017-0854-0)
Supplement: Supplementary file 2 — Table S1. List of primers for qPCR experiments. Table S2. Patient characteristics in prostate cancer cohort and the derived datasets. Table S3. Cox proportional hazard model for derivation dataset. Table S4. Cox proportional hazard model for validation dataset. (DOCX 115 kb) [file 12916_2017_854_MOESM2_ESM.docx]

| **Table S1. List of primers for qPCR experiments** | | |
| --- | --- | --- |
| **Gene Name** | **Forward primer(5’-3’)** | **Reverse primer(5’-3’)** |
| **Maspin** | **CTACTTTGTTGGCAAGTGGATGAA** | **ACTGGTTTGGTGTCTGTCTTGTTG** |
| **TMPRSS2** | **CTGGTGGCTGATAGGGGAT** | **GTCTGCCCTCATTTGTCGAT** |
| **KLK2** | **AGCCTGCCAAGATCACAGAT** | **GGAAGAACTCCTCTGGTTCG** |
| **FASN** | **AGGATCACAGGGACAACCTG** | **ACTCCACAGGTGGGAACAAG** |
| **FKBP5** | **TCCCTCGAATGCAACTCTCT** | **GCCACATCTCTGCAGTCAAA** |
| **PSA** | **AGGTCAGCCACAGCTTCCCA** | **GGGCAGGTCCATGACCTTCA** |
| **ING3** | **CAGCCAGTGAACAATCACCAT** | **CAGCACAGACACGTTCCTCT** |
| **GUSB** | **CGTCCCACCTAGAATCTGCT** | **TTGCTCACAAAGGTCACAGG** |
| **NKX3.1** | **GTACCTGTCGGCCCCTGAACG** | **GCTGTTATACACGGAGACCAGG** |
| **Actin** | **GAACCCTAAGGCCAACCGTGA** | **AGGAAGAGGATGCGGCAGTGG** |
| **Snail** | **ACTGCAACAAGGAATACCTCAG** | **GCACTGGTACTTCTTGACATCTG** |
| **Twist1** | **GTCCGCAGTCTTACGAGGAG** | **GCTTGAGGGTCTGAATCTTGCT** |
| **Ecadherin** | **ATTTTTCCCTCGACACCCGAT** | **TCCCAGGCGTAGACCAAGA** |

| **Table S2. Patient characteristics in prostate cancer cohort and the derived datasets** | | | |
| --- | --- | --- | --- |
| **Characteristic** | **Derivation Dataset** | **Validation Dataset** | **Combined** |
|  | **n=133** | **n=123** | **n=256** |
| **Age at diagnosis** | **55-97** | **54-96** | **54-97** |
| **Death** | **Survived: 107** | **Survived: 94** | **Survived: 200** |
|  | **PCa-Related death: 23** | **PCa-Related death: 22** | **PCa-Related death: 45** |
|  | **Missing: 3** | **Missing: 7** | **Missing: 10** |
| **CRPC** | **PCA: 100** | **PCA: 96** | **PCA: 195** |
|  | **CRPC: 32** | **CRPC: 27** | **CRPC: 59** |
|  | **Missing: 1** | **Missing: 1** | **Missing: 2** |
| **Gleason Score** | **=< 7 : 63** | **=< 7 : 60** | **=< 7 : 122** |
|  | **> 7 : 67** | **> 7 : 59** | **> 7 : 126** |
|  | **Missing: 3** | **Missing: 4** | **Missing: 7** |
| **AR expression** | **Low: 60** | **Low: 57** | **Low: 117** |
|  | **High: 73** | **High: 66** | **High: 138** |
| **ERG expression** | **= 0: 101** | **= 0: 88** | **= 0: 188** |
|  | **> 0: 29** | **> 0: 33** | **> 0: 62** |
|  | **Missing: 3** | **Missing: 2** | **Missing: 5** |
| **ING3 expression** | **< 1.66: 83** | **< 1.66: 72** | **< 1.66: 155** |
|  | **> 1.66: 50** | **> 1.66: 51** | **> 1.66: 101** |

| **Table S3. Cox proportional hazard model for Derivation dataset** | | | | | |
| --- | --- | --- | --- | --- | --- |
| **Covariate** | **Coefficient** | **SE** | **p-value** | **Hazard Ratio** | **95% CI** |
| **ING3** | **1.197** | **0.52** | **0.021** | **3.309** | **1.193-9.173** |
| **Age** | **-0.36** | **0.031** | **0.247** | **0.965** | **0.908-1.025** |
| **CRPC** | **1.778** | **0.541** | **0.001** | **5.915** | **2.047-17.090** |
| **Gleason Score** | **2.426** | **0.731** | **0.001** | **11.31** | **2.699-47.393** |
| **AR expression** | **-0.314** | **0.532** | **0.555** | **0.73** | **0.257-2.073** |
| **ERG expression** | **-1.768** | **0.697** | **0.011** | **0.171** | **0.044-0.669** |

| **Table S4. Cox proportional hazard model for Validation dataset** | | | | | |
| --- | --- | --- | --- | --- | --- |
| **Covariate** | **Coefficient** | **SE** | **p-value** | **Hazard Ratio** | **95% CI** |
| **ING3** | **0.944** | **0.471** | **0.045** | **2.571** | **1.022-5.468** |
| **Age** | **0.016** | **0.026** | **0.538** | **1.016** | **0.966-1.069** |
| **CRPC** | **1.067** | **0.494** | **0.031** | **2.906** | **1.102-7.659** |
| **Gleason Score** | **1.709** | **0.714** | **0.017** | **5.524** | **1.363-22.383** |
| **AR expression** | **0.549** | **0.534** | **0.305** | **1.731** | **0.607-4.934** |
| **ERG expression** | **0.556** | **0.535** | **0.299** | **1.743** | **0.611-4.976** |
